# Polymorphisms within Genes Coding for IL-17A and F and Their Receptor as Clinical Hallmarks in Ankylosing Spondylitis

**DOI:** 10.1155/2021/3125922

**Published:** 2021-10-27

**Authors:** Joanna Wielińska, Jerzy Świerkot, Katarzyna Kolossa, Bartosz Bugaj, Monika Chaszczewska-Markowska, Sławomir Jeka, Katarzyna Bogunia-Kubik

**Affiliations:** ^1^Laboratory of Clinical Immunogenetics and Pharmacogenetics, Hirszfeld Institute of Immunology and Experimental Therapy, Polish Academy of Sciences, R. Weigla 12, 53-114 Wroclaw, Poland; ^2^Department of Rheumatology and Internal Medicine, Wroclaw Medical University, Borowska 213, 50-556 Wroclaw, Poland; ^3^Department of Rheumatology and Connective Tissue Diseases, Jan Biziel University Hospital No. 2, Ujejskiego 75, 85-168 Bydgoszcz, Poland; ^4^Ludwik Rydygier Collegium Medicum in Bydgoszcz, Nicolaus Copernicus University in Torun, Jagiellońska 15, 85-067 Bydgoszcz, Poland

## Abstract

IL-17A and IL-17F together with their coreceptor (IL-17RA/RC) were reported to play a significant role in the pathogenesis of spondyloarthritis. The group of axial spondyloarthritis comprises ankylosing spondylitis (AS), a rheumatic disease characterized by chronic inflammation of the joints in the spine. This study is aimed at investigating *IL-17A*, *IL-17F*, *IL-17RA*, and *IL-17RC* polymorphisms as potential biomarkers of disease susceptibility, clinical parameters, and anti-TNF treatment outcome in a cohort of Polish ankylosing spondylitis patients. In total, 328 subjects, including 138 AS patients and 190 healthy volunteers, participated in the study. Genotyping of *IL-17A* rs2275913 (G/A), *IL-17F* rs763780 (A/G), *IL-17RA* rs4819554 (A/G), and *IL-17RC* rs708567 (G/A) was performed on real-time PCR instrument using LightSNiP assays. No significant differences were revealed in genotype and allele distribution between patients and controls despite the association of the *IL-17RC* rs708567 *AA* homozygosity with the earlier onset of the disease. Moreover, some relationships between *IL-17F* rs763780 and *IL-17RA* rs4819554 polymorphisms with clinical parameters related to the disease activity and anti-TNF treatment outcome were observed. *IL-17F* rs763780 G allele was found to be associated with high disease activity and BASDAI after 6 months and poor response to the treatment while higher VAS values were more common among *IL-17RA* rs4819554 G variant carriers. In conclusion, the *IL-17F* rs763780 polymorphism should be considered as a promising biomarker of disease activity and anti-TNF treatment outcome. The *IL-17RA* rs48419554 G allele may serve as a potential marker of disease severity in Polish AS patients.

## 1. Introduction

Ankylosing spondylitis (AS) is characterized by visible radiographic changes within the spine or sacroiliac joints. The axial spondyloarthritis (axSpA) group comprises AS, radiographic axial spondyloarthritis, and a nonradiographic (nr-axSpA) form of the disease [1]. AS patients suffer from inflammatory back pain and morning stiffness. Symptoms can also involve enthesitis and peripheral arthritis manifestations. The disease affects mostly men (ratio men to women is 2 to 1), those under thirty years of age, and with a strong genetic association linked to HLA-B27 [2]. Prevalence differs between geographical regions and ethnicity, reaching 0.23% in the general European population [3] and roughly 0.083% in Polish people [4].

The standard pharmacological treatment against AS involves tumour necrosis factor-alpha (TNF-*α*) inhibitor (anti-TNF) dosage after the primary failure of nonsteroidal anti-inflammatory (NSAIDs) administration. The long-term anti-TNF approach has positive effects on patient's functional outcome, lessens disease activity, and reduces radiographic progression [5].

The IL-17 family consists of six cytokines: IL-17A, IL-17B, IL-17C, IL-17D, IL-17E (IL-25), and IL-17F. Those proteins transmit signals through defined heterodimeric transmembrane receptors (IL-17R). IL-17A, IL-17F, and IL-17A/F heterodimer act via the IL-17RA/RC receptor complex. IL-17E triggers responses through IL-17RA/RB, and IL-17C induces the IL-17RA/RE heterodimer. For other proteins, the heterotrimeric receptor compound has not been fully elucidated. IL-17A and IL-17F have a high degree of homology, and both are secreted by Th17 cells, *γδ* T cells, innate lymphoid cells, cytotoxic T cells, and natural killer T (NKT) cells [6].

IL-17 was reported to have a crucial role in the immunopathogenesis of spondyloarthritis [7]. Elevated levels of IL-17 in serum have been observed in ankylosing spondylitis patients [8]. Besides, associations between IL-17 level and the Bath Ankylosing Spondylitis Disease Activity Index (BASDAI) [9, 10] have been described.

However, our knowledge of *IL-17* gene polymorphisms in AS is still limited. Thus, based on previous research focused on rheumatoid arthritis [11] and osteoarthritis [12], we hypothesized that *IL-17A* rs2275913 and *IL-17* rs763780 might influence AS susceptibility. The targeted single nucleotide polymorphisms were selected based on the available literature, especially on Caucasians, as well as our preliminary experiment on rheumatoid arthritis patients [13]. We also decided to investigate polymorphisms of IL-17 receptors *IL-17RA* and *IL-17RC*. *IL-17RA* rs4819554 was previously linked with response to etanercept in psoriatic arthritis [14], while *IL-17RC rs708567* was associated with lupus arthritis [15] and was described in Tunisians with rheumatoid arthritis [16]. Moreover, the newly performed study considered the *IL-17RA* polymorphism as an AS risk factor [17]. To the best of our knowledge, no investigations have been conducted to assess the association between *IL-17RA* and *IL-17RC* polymorphisms and rheumatic diseases in the Polish population.

This study examines the *IL-17A* rs2275913, *IL-17F* rs763780, *IL-17RA* rs4819554, and *IL-17RC* rs708567 genetic variants as potential biomarkers of disease susceptibility, clinical parameters, and anti-TNF treatment outcome in a cohort of Polish AS patients.

## 2. Materials and Methods

### 2.1. Patients and Controls

One hundred thirty-eight AS patients and one hundred ninety controls were involved in the study. AS patients were recruited from the Department of Rheumatology and Internal Medicine, Wroclaw Medical University, Poland, and from the Department of Rheumatology and Connective Tissue Diseases, Jan Biziel University Hospital No. 2 in Bydgoszcz, Poland. All the participants diagnosed with AS were Caucasians over 18 years of age, and 74% (102/138) were male. Included criteria comprise a resistance to treatment with at least two nonsteroidal antirheumatic drugs (NSAIDs), high disease activity before starting biological treatment, initialization of anti-TNF therapy at the time of the research, and complete medical history. Subjects with the coexistence of acute or chronic disorders besides AS, other autoimmune diseases, malignancies, or current infections, during pregnancy and breastfeeding, as well as with insufficient clinical records, and an unwillingness or inability to cooperate were excluded from the study.

AS patients were diagnosed according to the 1984 modified New York Criteria [18].

Data such as gender, age, disease onset, disease duration, body mass index (BMI), presence of HLA-B27, C-reactive protein (CRP) level, pain visual analogue scale (VAS, range: 0-100 mm), and Bath Ankylosing Spondylitis Disease Activity Index (BASDAI, range: 0-10) were collected from patients.

90.4% of patients were HLA-B27 positive, and most of them (76.7%) had the axial form of AS. Drug administration comprised MTX in 27.5% of cases, corticosteroids (20.3%) of subjects and NSAIDs have been taken by 71.3% of patients.

Bath Ankylosing Spondylitis Disease Activity Index (BASDAI) was used to calculate disease activity, which was considered to be high (BASDAI ≥ 4), moderate (3 ≤ BASDAI < 4), or low (BASDAI < 3). The clinical outcome was assessed after 3 and 6 months of anti-TNF treatment. Significant improvement after therapy was defined as a reduction of BASDAI (ΔBASDAI ≥ 2.0), good outcome as ΔBASDAI ≥ 2.0 and BASDAI < 3.0 at the endpoint, moderate response as ΔBASDAI ≥ 2.0 and BASDAI ≥ 3.0 at the endpoint, and no improvement as ΔBASDAI < 2.0 [19].

The patient's demographic and clinical characteristics are described in Table 1. The data are presented as median with range (minimum to maximum).

The control group was enrolled from the healthy volunteers, 63 females (33%) and 127 (67%) males, from the Regional Centre of Transfusion Medicine and Blood Bank in Wroclaw without a personal history of rheumatic diseases.

Informed consent was obtained from all participants involved in the study. The research was approved by the Wroclaw Medical University Ethics Committee (identification code KB-625/2016).

### 2.2. SNP Selection and Genotyping

Tested genetic variants were selected based on analysis of previous publications and search results from NCBI Database of Short Genetic Variations (dbSNP) and SNPinfo Web Server [20]. Minor allele frequency (MAF) in EUR was above 5% (1000 Genomes Project) [21].

In total, four single nucleotide polymorphisms (SNPs) were chosen for analysis: *IL-17A* rs2275913 (G/A) and *IL-17F* rs763780 (A/G) located on chromosome 6, *IL-17RA* rs4819554 (A/G) located on chromosome 22, and *IL-17RC* rs708567 (G/A) located on chromosome 3. Two of them, *IL-17F* rs763780 and *IL-17RC* rs708567 are missense variants in exon 3 (His161Arg) and exon 4 (Ser111Leu), respectively. *IL-17A* rs2275913 and *IL-17RA* rs4819554 are substitutions within gene promoter regions with a predicted transcription factor binding site (TFBS).

Whole blood samples were collected in EDTA tubes (BD Vacutainer® Blood Collection Tubes). Genomic DNA was isolated from peripheral blood using QIAamp DNA Blood Midi/Maxi Kit (Qiagen, Hilden, Germany) following the manufacturer's protocol. The genotyping of selected SNPs: *IL-17A* rs2275913 (G/A), *IL-17F* rs763780 (A/G), *IL-17RA* rs4819554 (A/G), and *IL-17RC* rs708567 (G/A) was performed using LightSNiP assays (TIB MOLBIOL, Berlin, Germany) on the LightCycler 480 Real-Time PCR Instrument (Roche Diagnostics, Basel, Switzerland).

### 2.3. Statistical Analysis

The genotype frequencies were tested for the Hardy-Weinberg equilibrium (HWE). Potential differences in allele and genotype distributions between the patient and control groups were calculated using Fisher's exact test. Continuous variables were tested for normal distribution by the Shapiro–Wilk test. Quantitative variables that were normally distributed were presented as mean ± SEM, while medians with interquartile ranges (IQRs) were calculated for nonnormally distributed variables. The unpaired two-sample Wilcoxon test (for nonparametric data) or unpaired two-sample *t*-test (for normally distributed data) were performed to identify associations within genetic variants and clinical parameters. Fisher's exact test was also applied to detect relationships between genotypes and categorical variables such as disease activity or treatment outcome. A *p* value lower than 0.05 (*p* < 0.05) was considered statistically significant. All statistical analysis was performed using R Software (http://www.r-project.org) and GraphPad Prism 7 for Windows.

## 3. Results

### 3.1. Distribution of IL-17A, IL-17F, IL-17RA, and IL-17RC Alleles and Genotypes in Patients and Controls

The distribution of genotypes and alleles of *IL-17A* rs2275913, *IL-17F* rs763780, *IL-17RA* rs4819554, and *IL-17RC* rs708567 did not differ between AS patients and healthy individuals (Table 2). Also, no significant gender-dependent differences were detected between patients and healthy subjects (not shown). Please note that none of the patients or controls were homozygous for the *IL-17F* rs763780 G allele. Thus, in the further analyses, *AA* homozygotes were being compared with *AG* genotype reflecting also the *G* allele carriers.

On the other hand, the significant association between disease onset and genotype frequency was observed for *IL-17RC* rs708567 SNP. Patients with *AA* genotype had a lower age of disease onset (29.39 ± 1.405) than those with *G* allele (33.43 ± 1.001) (*AA* vs. *AG+GG*, *p* = 0.022; *AA* vs. *AG*, *p* = 0.015). However, disease duration was not found to be affected by any of the analysed SNPs.

### 3.2. Associations between *IL-17A*, *IL-17F*, *IL-17RA*, and *IL-17RC* Genotypes and Clinical Parameters

The potential associations between *IL-17A*, *IL-17F*, *IL-17RA*, and *IL-17RC* genotypes and CRP level, VAS, and BASDAI values were analysed.

During anti-TNF therapy, these major clinical parameters were decreased. CRP level was significantly lower after 3 and 6 months compared to baseline (*p* < 0.0001). Also, VAS and BASDAI were reduced after 3 and 6 months related to baseline, as well as after 6 months in comparison to 3 months of therapy (*p* < 0.0001) (Table 1). A significant improvement in clinical parameters was achieved after administration of anti-TNF agents.

Higher VAS values at the baseline were found in *IL-17A* rs2275913 *GG* (*GG* vs. *GA+AA*, *p* = 0.005; *GG* vs. *GA*, *p* = 0.006) and *IL-17F* rs763780 *AG* (*AA* vs. *AG*, *p* = 0.027) genotype carriers (Table 3(a)).

The *IL-17RA* rs4819554 G allele was found to be more common among patients who presented with higher VAS and BASDAI values after anti-TNF treatment induction.

Patients possessing the *IL-17RA* rs4819554 G allele had higher VAS values after 3 months of anti-TNF therapy (*AA* vs. *AG+GG*, *p* = 0.002). This result was also observed after 6 months of treatment (*AA* vs. *AG+GG*, *p* = 0.002).

Besides, *IL-17RA* rs4819554 G patients demonstrated greater BASDAI values at 6 months of therapy than *AA* homozygotes (*AA* vs. *AG+GG*, *p* = 0.046) (Table 3(b)). The *IL-17RA* results concerning VAS at 3 and 6 months and BASDAI at 6 months were confirmed by the overdominant model (*AA+GG* vs. *AG*, *p* = 0.008, *p* = 0.006, and *p* = 0.045, respectively). Additionally, a significant relationship with an absolute BASDAI change (*Δ*BASDAI 0-3 m.) (*AA+GG* vs. *AG*, *p* = 0.027) was noted. Tendencies were observed regarding BASDAI score at 3 months (*AA* vs. *AG+GG*, *p* = 0.066; AA+GG vs. AG, *p* = 0.064) and absolute BASDAI change (*Δ*BASDAI 0-6 m.) (*AA+GG* vs. *AG*, *p* = 0.057).

The BASDAI parameter at 6 months was also higher in the group of AS individuals bearing *IL-17F* rs763780 *AG* genotype (*G* allele) (*AA* vs. *AG*, *p* = 0.035) in comparison to *AA* carriers (Table 3(a)).

As for CRP levels, *IL-17RC* rs708567 and *IL-17A* rs2275913 polymorphisms were identified as significantly associated with CRP level after 3 months of TNF inhibitor administration. At that time point, *IL-17A* rs2275913 *GG* was more frequently observed among patients with an elevated level of CRP (>10 mg/l) (*GG* vs. *GA+AA*, *p* = 0.022, OR = 3, and 95%CI = 1.237‐7.046), and *IL-17RC* rs708567 G patients showed a significantly higher CRP level as compared to the *AA* patients (*AA* vs. *AG+GG*, *p* = 0.043; *AA* vs. *AG*, *p* = 0.018) (Table 3(b)).

No other significant differences between clinical parameters of AS patients and their *IL-17* genotype distribution were detected.

### 3.3. Effect of IL-17F Polymorphisms on the Disease Activity and Anti-TNF Treatment Outcome

Before the anti-TNF administration, all patients were characterized with high disease activity (BASDAI > 4). After 3 months of therapy, 25.4% (35/138), 44.9% (62/138), and 29.7% (41/138) of subjects had a high, moderate, and low disease activity, respectively. After 6 months, only 3.03% (4/132) and 1.52% (2/132) were described with high and moderate disease activity, respectively. The remaining 95.5% (126/132) of patients presented low disease activity. After 3 months of anti-TNF treatment, 97.8% (135/138) achieved a good or moderate outcome, and 2.2% (3/128) were nonresponders. Similarly, after 6 months, 3% (4/132) of patients did not respond positively to treatment.

Out of *IL-17A*, *IL-17F*, *IL-17RA*, and *IL-17RC* polymorphisms studied, a significant association concerning disease activity was detected for the *IL-17F* rs763780 variant. AS patients homozygous for the *A* allele more likely presented low or moderate disease activity (BASDAI < 4) after 6 months of treatment than heterozygotes (*AA* vs. *AG*, *p* = 0.035, OR = 13.22, and 95%CI = 1.82‐87.84). The same genotype was significantly more common among subjects with a good or moderate response to TNF inhibitor therapy (*AA* vs. *AG*, *p* = 0.035, OR = 13.22, and 95%CI = 1.82‐87.84).

The other studied *IL-17A* rs2275913, *IL-17RA* rs4819554, and *IL-17RC* rs708567 genetic variants were not found to significantly differ among AS patients in respect to disease activity and biological agent treatment outcome.

## 4. Discussion

In the present study, patients with ankylosing spondylitis and controls were genotyped for the *IL-17A*, *IL-17F*, *IL-17RA*, and *IL-17RC* polymorphisms to assess whether their genetic variants may be associated with susceptibility to the disease, clinical parameters, and anti-TNF treatment outcome in our Polish population.

Comparison made between our patients and controls did not show any significant association with predisposition to the disease as in both groups, similar distributions of alleles and genotypes of all SNPs studied were observed. However, disease onset was found to be affected by the *IL-17RC* rs708567 SNP and the presence of *AA* homozygosity prevailed in patients that had a lower age of disease onset than those with *G* allele.

Among currently analysed genetic variants, *IL-17A* rs2275913 and *IL-17F* rs763780 have been previously extensively studied for associations with various rheumatic disorders.

In our current study, neither *IL-17A* rs2275913 nor *IL-17F* rs763780 was found to be associated with AS risk. Nevertheless, association with AS susceptibility and *IL-17A* rs2275913 in Chinese [22] and *IL-17F* rs763780 in Turkish [23] populations has been reported. Erkol et al. did not find the relationship between *IL-17A* rs2275913 and AS susceptibility in Turkish patients [23]. More recently, Rocha Loures et al. reported rs2275913 *A* variant and rs763780 G allele as risk factors for AS, spondyloarthritis (SpA), and psoriatic arthritis (PsA) in Brazilian patients [24].

As for the associations of *IL-17* polymorphisms with other diseases, many previous studies focused on osteoarthritis (OA) and rheumatoid arthritis (RA). Results of analysis performed on an Asian OA group suggested that the *IL-17A* rs2275913 *A* allele [25–27] and *IL-17F* rs763780 G variant [26] increased susceptibility to knee OA. In Caucasians, no association between *IL-17A* rs2275913 polymorphism and risk of hip or knee OA was found [28], but the *IL-17F* rs763780 G allele had a significant impact on the risk of the hip [28] and knee OA [29]. *IL-17A* rs2275913 *GA* [29], *IL-17F* rs763780 *AA* [28], and *IL-17A-F* G-A haplotype [30] seem to play a rather protective role in the knee, hip, or hip and knee OA, respectively. Further meta-analysis performed by Lu et al. highlighted higher susceptibility to OA in patients with *IL-17A* rs2275913 *A* allele and *IL-17F* rs763780 G allele among Asians, as well as with *IL-17F* rs763780 *G* genetic variant in a Caucasian cohort [12].

Interestingly, papers concerning the role of *IL-17A* rs2275913 and *IL-17F* rs763780 in RA are inconsistent. *IL-17A* rs2275913 *GG* genotype [31, 32] and *G* allele [33] have been found to increase susceptibility to RA, whereas Shen et al. described *AA* genotype as being linked to lower RA risk [34]. Other studies showed no significant correlations between *IL-17A* rs2275913 variant and prevalence to develop RA in Polish [13, 35], Turkish [36], Brazilian [37], Tunisian [16, 38], Algerian [39], Mexican [40], and Egyptian [41] patients. Growing evidence suggests that *IL-17F* rs763780 G is associated with susceptibility to the disease [13, 33, 38]. However, many studies did not confirm this polymorphism as an RA risk factor [35–37, 39, 41, 42]. Recent meta-analysis findings led to the identification of *IL-17A GG* and *IL-17F AG* genotypes as more frequently distributed among RA patients [11].

With regard to *IL-17RA* and *IL-17RC* polymorphisms, the present analysis did not show differences in genotype and allele distribution between patients and controls. This observation confirms previous results for *IL-17RC* among RA patients [16] but stays in contrast with *IL-17RA* developments in AS Spanish cohorts [17]. *IL-17RA* rs48419554 was also identified as a risk factor for psoriasis [43, 44]. However, other *IL-17RA* SNPs were not found to be associated with PsA [45].

Our current results also show that *IL7RC* rs708567 G variant has an effect on disease onset and is more frequently detected among patients that developed the diseases approximately 4 years later than *AA* homozygotes. To the best of our knowledge, no one has studied *IL-17RC* rs708567 in AS so far. This genetic variant and its homozygosity were also described to affect arthritis among systemic lupus erythematosus Bulgarian patients [46]. On the other hand, in a study conducted by Dhaouadi et al., *IL7RC A* allele of this polymorphism tended to show higher DAS28 in RA subjects [16]. However, the functional consequence of *IL-17RC* rs708567 polymorphism remains unknown.

In the present study, some interesting results were described regarding the *IL-17F* rs763780 SNP and our cohort of patients with AS and unfavourable effect of the *IL-17F* rs763780 G allele.

Likewise, the *IL-17F* rs763780 *G* allele was observed by Paradowska-Gorycka et al. to be positively correlated with the number of tender joints, as well as to tend to reach insignificantly higher values of DAS28-CRP and health assessment questionnaire (HAQ) score [42].

According to earlier findings established in Turkey, the *IL-17F* rs763780 *GG* genotype was prone to greater BASFI scores and *AG* variant to higher CRP level [23]. As suggested, the evidence we found points to an association between this polymorphism and disease activity in AS patients. Our results show that the *AG* genotype is significantly correlated with higher, both VAS values before treatment and BASDAI score after 6 months. Also, we link *AA* genotype with moderate/low disease activity and good/moderate response to treatment after 6 months. This concurs well with results obtained by Prieto-Peréz et al., who observed that rs763780 can predict response to adalimumab at 6 months, in psoriasis [47].

Of note, our previous analysis showed the association of the *IL-17F* rs763780 G allele with higher IL-17F secretion [48]. Also, Braga et al. observed this association between the *IL-17F* rs763780 *G* allele and increased IL-17F serum levels in Brazilian AS patients and controls [49]. These results suggest that alleles or genotypes associated with higher IL-17F production may play an unfavourable role.

Recently, the novel insight into functional consequences of the *IL-17F* polymorphism was described by Nisar et al. The change at position 161 (His to Arg) is located in the C-terminal end of IL-17, which interacts with IL-17RA. This substitution resulted in more favourable conformation, enhanced stability of the trimeric IL-17A/F/IL-17RA complex. The stronger binding may induce the proinflammatory effect and influence the severity of RA [50].

One of our previous studies performed on RA patients found that *IL-17A* rs2275913 *GG* homozygous females were characterized with the most active disease after 3 months and poor response to anti-TNF therapy [13]. On the other hand, de la Peña et al. reported that *A* allele carriers were predicted to present more severe RA and needed more than three DMARDs to control the disease [51].

It has been reported that the *IL-17A* rs2275913 polymorphism located in the promoter region can regulate gene transcription and stimulate IL-17 cytokine secretion (-197A allele) [52].

Current analysis demonstrates a significant correlation between the *IL-17A GG* genotype and higher VAS values before starting therapy in AS patients. The same genotype more frequently characterized patients with elevated CRP after 3 months. We did not find any significant correlation between *IL-17A* variants and response to the therapy. However, it was reported in the literature that rs2275913 was associated with response to anti-TNF therapy among patients with inflammatory bowel disease [53].

Our results are also in line with the findings of Vidal-Castiñeira et al. concerning significantly higher BASDAI scores in AS patients carrying the *IL-17RA* rs4819554 G allele. Moreover, our study reveals associations between the *IL-17A G* allele and greater VAS after 3 and 6 months of anti-TNF treatment. These correlations are worth mentioning because they indicate an impact of *G* variant, located in the gene promoter, on AS severity. This region was also noted to affect the response to anti-TNF therapy outcome in psoriasis [54].

The *IL-17RA* rs4819554 variant is encoded within the promotor region and may have a functional effect by modulating the gene transcription. This SNP was found to be in linkage disequilibrium with rs4819553 and rs4819958. Those polymorphisms are predicted to be related to transcription factor binding sites (TFBSs) belonging to the Ikaros (IK) family. These are involved in Th17 cell differentiation [54].

In *G* allele carriers, the increase of Th17 cytokines could promote the pathogenic mechanism via IL-23/Th17 pathway [54]. It could explain the higher VAS values and BASDAI scores that we observed.

These findings shed some light on common genetic variants in *IL-17A*, *IL-17F*, and *IL-17RA* genes. The investigated polymorphisms can affect biological activity of the protein and thus influence immunological features like a response to etanercept [14].

There is still considerable controversy surrounding *IL-17* SNP relationships and AS development. Therefore, our results need to be interpreted with caution. In fact, population diversity and treatment approach may explain the differences between studies. Although the advantage of our methodology is homogeneity of the Polish population, we are aware that the main limitation of our study is the relatively limited number of cases included in the analysis. Therefore, further data collection from AS patients is required to confirm these observations.

Other interesting genetic variants within *IL-17F* include rs11465553 [35] and rs2397084 [33, 35, 36, 38, 39, 42], which were investigated in RA. In a Polish cohort, the rs2397084 polymorphism was correlated with longer disease duration [42], whereas in Tunisians, it was associated with disease severity [38]. Additionally, rs2397084 [29] and rs1889570 [28, 30] were studied in osteoarthritis patients. Regarding the *IL-17A* gene, rs3804513 was associated with radiographic progression in early RA [55]. Other *IL-17A* polymorphisms were studied in a Chinese population. *IL-17A* rs4711998 and rs8193037 were not associated with RA, whereas rs3819024, rs3819025, and rs8193036 were correlated with the risk of RA [34]. These polymorphisms may be of interest for further study on ankylosing spondylitis.

## 5. Conclusions

The analysis shows that *IL-17* polymorphisms are associated with clinical parameters in Polish patients with ankylosing spondylitis and have influence on AS severity and potential course of the disease and may be biomarkers of response to anti-TNF drugs in Polish patients. The *IL-17F* rs763780 polymorphism should be considered as a candidate biomarker of disease activity and anti-TNF treatment outcome. The *IL-17RA* rs48419554 G allele may serve as a potential marker of disease severity.

## Figures and Tables

**Table 1:** Clinical characteristics of the study cohort.

Characteristic	*N*	Median (range)
Age (years)	138	43.5 (22-75)
Disease duration (years)	135	10 (0-48)
Disease onset (years)	135	33 (6-56)
BMI	113	25.32 (18.61-40.31)
CRP before treatment (mg/l)	108	16.83 (0.3-561)
CRP at 3 months (mg/l)	79	5.75 (0.2-175)^∗^
CRP at 6 months (mg/l)	72	5.495 (0.2-204.3)^∗^
VAS before treatment (mm)	132	80 (45-100)
VAS at 3 months (mm)	138	30 (0-80)^∗^
VAS at 6 months (mm)	131	20 (0-100)^∗^
BASDAI before treatment	138	8 (4.05-10)
BASDAI at 3 months	138	3.2 (0.7-6.7)^∗^
BASDAI at 6 months	132	2.25 (0.2-9.75)^∗^
Treatment (anti-TNF drug)	*N* = 138	*n* (%)
Adalimumab		63 (45.6%)
Etanercept		44 (31.9%)
Certolizumab		17 (12.3%)
Golimumab		12 (8.69%)
Infliximab		2 (1.45%)

*N*: number of patients with clinical information; BMI: body mass index; CRP: C-reactive protein; MTX: methotrexate; NSAIDs: nonsteroidal anti-inflammatory drugs; BASDAI: Bath Ankylosing Spondylitis Disease Activity Index; VAS: visual analogue scale; HLA-B27: human leukocyte antigen B27. ^∗^*p* < 0.001; *p* value comparing the clinical variables between baseline and after 3 or 6 months of treatment.

**Table 2:** The distribution of *IL-17* genotypes and alleles in AS patients and the control group.

	Patients	Controls
*IL-17A* rs2275913	*N* = 138	*N* = 190
*G*	174 (63.0%)	234 (61.6%)
*A*	102 (37.0%)	146 (38.4%)
*GG*	50 (36.2%)	69 (36.3%)
*GA*	74 (53.6%)	96 (50.5%)
*AA*	14 (10.1%)	25 (13.2%)
*IL-17F* rs763780	*N* = 138	*N* = 189
*A*	265 (96.0%)	359 (95.0%)
*G*	11 (4.0%)	19 (5.0%)
*AA*	127 (92.0%)	170 (89.9%)
*AG*	11 (7.97%)	19 (10.1%)
*GG*	0 (0%)	0 (0%)
*IL-17RA* rs4819554	*N* = 138	*N* = 190
*A*	215 (77.9%)	311 (81.8%)
*G*	61 (22.1%)	69 (18.2%)
*AA*	83 (60.1%)	126 (66.3%)
*AG*	49 (35.5%)	59 (31.1%)
*GG*	6 (4.35%)	5 (2.63%)
*IL-17RC* rs708567	*N* = 138	*N* = 189
*A*	150 (54.3%)	205 (54.2%)
*G*	126 (45.7%)	173 (45.8%)
*AA*	41 (29.7%)	47 (24.9%)
*AG*	68 (49.3%)	111 (58.7%)
*GG*	29 (21.0%)	31 (16.4%)

**(a):** 

	*IL-17A* rs2275913						*IL-17F* rs763780			
	*GG*		*GA*		*AA*		*AA*		*AG*	
	*N*	Median (IQR)	*N*	Median (IQR)	*N*	Median (IQR)	*N*	Median (IQR)	*N*	Median (IQR)
CRP 0 m.	43	13.99 (7.06-47.09)	54	18 (6.983-38.86)	11	15.3 (7.85-33)	97	18.33 (7.825-39.27)	11	9.38 (5-16.73)
CRP 3 m.	30	10.11 (1.39-21.12)	40	5.1 (1.325-10.08)	9	2.6 (0.95-7.9)	71	6.2 (1.52-13.55)	8	1.3 (0.375-11.84)
CRP 6 m.	25	7.06 (0.8-12.67)	39	5.41 (0.9-15.6)	8	3.35 (0.9-9.758)	64	5.71 (1.55-13.22)	8	1.1 (0.35-19.76)
VAS 0 m.	48	87 (80-90)^(a)^	70	80 (70-90)	14	80 (70-90)	122	80 (72.5-90)^(b)^	10	88.5 (84.5-92.5)
VAS 3 m.	50	35 (24.5-40)	74	30 (29.5-40)	14	30 (25-30)	127	30 (27-40)	11	30 (22-35)
VAS 6 m.	47	20 (10-29)	71	20 (15-29)	13	20 (16-23.5)	120	20 (14.25-25)	11	20 (11-30)
BASDAI 0 m.	50	7.95 (7-9)	74	7.95 (6.775-8.6)	14	8.3 (6.375-9)	127	8 (6.8-8.8)	11	8.6 (7-9)
BASDAI 3 m.	50	3.275 (2.744-4.1)	74	3.2 (2.65-3.94)	14	3 (2.8-3.063)	127	3.2 (2.75-4)	11	3 (2.725-3.3)
BASDAI 6 m.	48	2.35 (1.925-2.8)	71	2.25 (2-2.7)	13	2 (2-2.825)	121	2.15 (2-2.7)^(c)^	11	2.5 (2.3-2.9)

**(b):** 

	*IL-17RA* rs4819554						*IL-17RC* rs708567					
	*AA*		*AG*		*GG*		*AA*		*AG*		*GG*	
	*N*	Median (IQR)	*N*	Median (IQR)	*N*	Median (IQR)	*N*	Median (IQR)	*N*	Median (IQR)	*N*	Median (IQR)
CRP 0 m.	63	16.93 (6.9-39.11)	41	18.46 (7.825-42.09)	4	9.62 (9.248-25.34)	32	14.8 (6.928-28.73)	54	18.4 (8.29-45.73)	22	14.12 (7.17-33.51)
CRP 3 m.	46	5.745 (1.3-13.78)	31	6.54 (1-12.27)	2	5.51 (1.52-9.5)	26	2.15 (0.675-10.53)^(g)^	40	9.46 (2.55-14.79)	13	3 (0.9-9.555)
CRP 6 m	44	6.49 (0.75-18.33)	26	4.655 (2.05-12.07)	2	5 (3.8-6.2)	23	3.6 (0.7-12.88)	34	6.06 (1.35-13.37)	15	4.2 (2-15.94)
VAS 0 m.	80	80.5 (80-90)	46	80 (70-90)	6	84.5 (68.25-91.25)	39	80 (70-90)	65	81 (79-90)	28	84 (76-90)
VAS 3 m.	83	30 (23-40)^(d)^	49	35 (30-40)	6	38 (25-46.25)	41	30 (25-38.5)	68	30 (26.5.5-40)	29	35 (30-40)
VAS 6 m.	81	20 (10-23.5)^(e)^	45	20 (20-30)	5	21 (15-35)	40	20 (11.25-26.5)	62	20 (15-25)	29	20 (10-30)
BASDAI 0 m.	83	8 (7-8.8)	49	7.9 (6.6-8.95)	6	8.213 (6.675-9.25)	41	7.8 (6.1-8.6)	68	7.95 (7-8.8)	29	8.2 (7.4-9)
BASDAI 3 m.	83	3.1 (2.55-3.7)	49	3.325 (3-4)	6	3.3 (1.975-4.2)	41	3 (2.175-3.95)	68	3.2 (3-4)	29	3.2 (2.5-3.9)
BASDAI 6 m.	81	2.1 (1.925-2.6)^(f)^	46	2.375 (2-2.8)	5	2.3 (1.45-2.9)	40	2.2 (2-2.5)	63	2.25 (2-2.8)	29	2.3 (1.95-2.8)

^(a)^ *GG* vs. *GA+AA*, *p* = 0.005; *GG* vs. *GA*, *p* = 0.006; *GA* vs. *GG+AA*, *p* = 0.016; ^(b)^*AA* vs. *AG*, *p* = 0.027; ^(c)^*AA* vs. *AG*, *p* = 0.035; ^(d)^*AA* vs. *AG+GG*, *p* = 0.002; *AA* vs. *AG*, *p* = 0.004; *AA+GG* vs. *AG*, *p* = 0.008; ^(e)^*AA* vs. *AG+GG*, *p* = 0.002; *AA* vs. *AG*, *p* = 0.004; *AA+GG* vs. *AG*, *p* = 0.006; (f) *AA* vs. *AG+GG*, *p* = 0.046; *AA* vs. *AG*, *p* = 0.040; *AA+GG* vs. *AG*, *p* = 0.045; ^(g)^*AA* vs. *AG+GG*, *p* = 0.043; *AA* vs. *AG*, *p* = 0.018; *AA+GG* vs. *AG*, *p* = 0.011. *N*: number of patients in groups; IQR: interquartile range; CRP: C-reactive protein; VAS: visual analogue scale; DAS28: disease activity score 28; BASDAI: Bath Ankylosing Spondylitis Disease Activity Index; 0 m.: at the baseline; 3 m.: after 3 months of therapy; 6 m.: after 6 months of treatment.

## Data Availability

The data that support the findings of this study are available from the corresponding author upon reasonable request.
